# Alternate transcription of the Toll-like receptor signaling cascade

**DOI:** 10.1186/gb-2006-7-2-r10

**Published:** 2006-02-17

**Authors:** Christine A Wells, Alistair M Chalk, Alistair Forrest, Darrin Taylor, Nic Waddell, Kate Schroder, S Roy Himes, Geoffrey Faulkner, Sandra Lo, Takeya Kasukawa, Hideya Kawaji, Chikatoshi Kai, Jun Kawai, Shintaro Katayama, Piero Carninci, Yoshihide Hayashizaki, David A Hume, Sean M Grimmond

**Affiliations:** 1 Eskitis Institute for Cell and Molecular Therapies, School of Biological and Biomedical Sciences, Griffith University, Brisbane 4111, Australia; 2 The Institute for Molecular Biosciences, The University of Queensland, Brisbane 4072, Australia; 3 Karolinska Institutet, S-171 77 Stockholm, Sweden; 4 Genome Exploration Research Group (Genome Network Project Core Group), RIKEN Genomic Sciences Center, RIKEN Yokohama Institute, Yokohama, Kanagawa 230-0045, Japan; 5 Genome Science Laboratory, Discovery Research Institute, RIKEN Wako Institute, Wako, Saitama 351-0198, Japan; 6 The Special Research Centre for Functional and Applied Genomics, The University of Queensland, St Lucia, 4072, Australia

## Abstract

**Background:**

Alternate splicing of key signaling molecules in the Toll-like receptor (Tlr) cascade has been shown to dramatically alter the signaling capacity of inflammatory cells, but it is not known how common this mechanism is. We provide transcriptional evidence of widespread alternate splicing in the Toll-like receptor signaling pathway, derived from a systematic analysis of the FANTOM3 mouse data set. Functional annotation of variant proteins was assessed in light of inflammatory signaling in mouse primary macrophages, and the expression of each variant transcript was assessed by splicing arrays.

**Results:**

A total of 256 variant transcripts were identified, including novel variants of Tlr4, Ticam1, Tollip, Rac1, Irak1, 2 and 4, Mapk14/p38, Atf2 and Stat1. The expression of variant transcripts was assessed using custom-designed splicing arrays. We functionally tested the expression of Tlr4 transcripts under a range of cytokine conditions via northern and quantitative real-time polymerase chain reaction. The effects of variant Mapk14/p38 protein expression on macrophage survival were demonstrated.

**Conclusion:**

Members of the Toll-like receptor signaling pathway are highly alternatively spliced, producing a large number of novel proteins with the potential to functionally alter inflammatory outcomes. These variants are expressed in primary mouse macrophages in response to inflammatory mediators such as interferon-γ and lipopolysaccharide. Our data suggest a surprisingly common role for variant proteins in diversification/repression of inflammatory signaling.

## Abstract 2

A systematic analysis of the FANTOM3 mouse cDNA dataset provides transcriptional evidence of widespread alternate splicing in the Toll-like receptor signaling pathway.

## Background

Infectious diseases have exerted enormous pressures on mammalian populations; this can be observed in the way in which innate immune systems have evolved to recognize a vast and rapidly changeable pathogen world. An effective innate immune system must not be restricted to the recognition of individual disease agents, but rather the molecular patterns associated with different classes of pathogens (pathogen-associated molecular patterns (PAMPs)), hence the evolution of pattern recognition receptors [1]. These receptor complexes are characteristically large, multimeric and polymorphic in the extracellular domains, and signal through highly conserved pathways to induce an acute inflammatory response.

The Toll-like receptors (Tlrs) are a highly evolutionarily conserved family of pattern recognition receptors, consisting of at least 13 members that are central to the recognition of a large collection of PAMPs (for review, see [2]). Tlr members have variable leucine-rich repeat extracellular domains and a characteristic toll-interleukin receptor (TIR) intracellular domain that signals through the highly conserved myeloid differentiation primary-response gene 88 (Myd88)/IL-1 receptor associated kinase (Irak)/tumor necrosis factor (TNF) receptor-associated factor-6/nuclear factor-κB cascade [3,4]. Mutations in various Tlrs have been clinically associated with susceptibility to infectious diseases, and Tlr members have been linked to chronic inflammatory diseases such as arteriosclerosis, periodontal diseases, arthritis, and lung disease (for review, see [5]). Pattern recognition receptors such as the Tlr family are essential for the rapid recognition of pathogens; equally important is an appropriate inflammatory response, central to which is the resolution of that inflammatory cascade (for review, see [6]).

A diverse repertoire of innate immune responses is vital to the survival of a population threatened by infectious disease, and those pathogens that exploit stereotyped host responses are among the most clinically devastating [7]. The high degree of signaling conservation within the TIR superfamily of receptors, even across phyla, may seem counter-intuitive, given the drive to diversify an immune response. Some specificity is determined within the Tlr family by the differential use of adapter proteins; Myd88 dependant and independent signaling events to some degree drive the recruitment of mitogen-activated protein kinases (MAPK), interferon, and protein kinase C pathways to the immune response [4,8,9]. Products of the inflammatory cascade such as IL-1 and TNF-α further amplify the inflammatory response [10].

From a genomic perspective, protein diversity is generated through the use of alternate exons from a transcriptional framework (TK) - on average three different proteins from each TK [11]. Concomitantly, generation of variant proteins is predicted to alter the signaling cascades that they participate in. The role of alternate splicing in the innate immune system is particularly interesting. It has been known for some time that type-1 interferon signaling is modified by variations in the type 1 interferon receptor (IFNAR)2. The short chain variant and the soluble form act as dominant negative proteins, modulating type I interferon responses [12-14]. The observation of dominant-negative variants of key Tlr signaling components includes Myd88s [15] and Irak2 [16]. These proteins are induced by Tlr signaling, and are necessary for resolution of a Tlr-directed immune response. This suggests that alternate splicing occurs in response to signal transduction pathways, and requires inducible recruitment of splice factors as well as tissue-specific splicing regulators.

Analysis of alternate splicing in the innate immune system has thus far been done on gene-by-gene basis (for review, see [17]). We surveyed the combined FANTOM3 fl-CDNA and public expressed sequence tag data set and identified a suite of novel transcripts predicted to alter signaling in the Tlr pathway. This study evaluates the variation in proteins arising from these alternate splicing events using a systematic bioinformatics approach; predicts the impact a variant protein will have on signal transduction in the Tlr signaling cascade, and tests the expression of these novel proteins in a model of inflammatory macrophage activation.

## Results and discussion

### Identification and validation of variant members of the Tlr signaling pathway

The FANTOM3 TK defined the start, end, and splice boundaries of all of the variant transcripts arising from a gene [11]. Seventy TKs were identified as generating two or more protein variants, and a total of 256 proteins were consequently associated with the Tlr pathway (an average of three protein products/TK). Each framework was reviewed for variant protein domains in order to predict the functional properties of those variants.

TKs were built for 106 members of the Tlr and c-Jun N-terminal kinase/p38 Mapk pathways, identified from the Kyoto Encyclopaedia of Genes and Genomes [18], plus additional scaffolding proteins and factors known to be important in macrophage biology [19]. The pathway members, TK identifiers, protein domain, and variant transcript data are available online [20].

**Figure 1:**
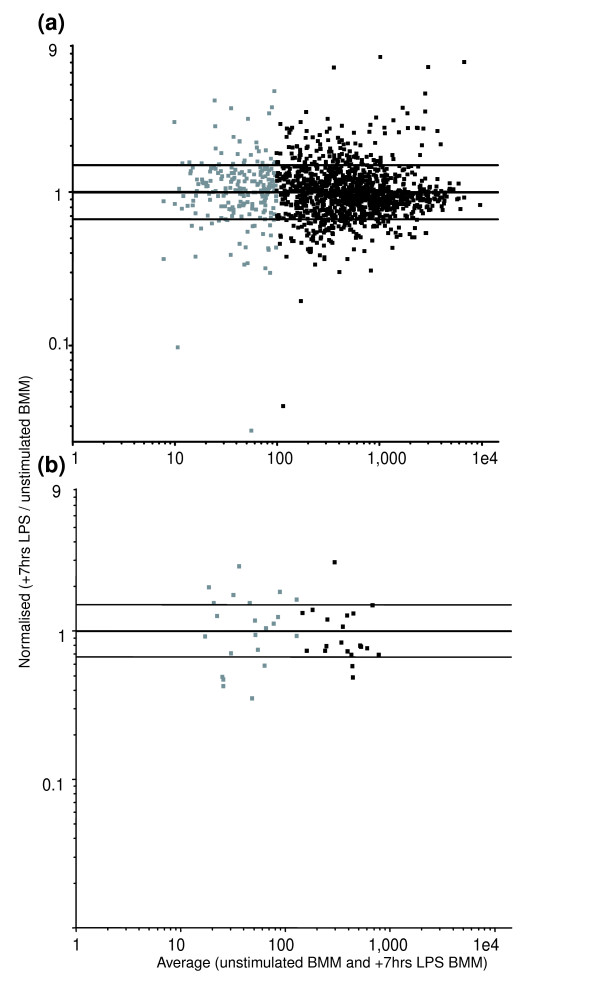
Distribution of signals across all microarray probes. The scatter plots show the distribution of signals from **(a) **junction and exon probes and **(b) **intron probes. The average signal of each probe (unstimulated bone-marrow derived macrophages (BMMs) and BMMs subjected to seven hours of stimulation with lipopolysaccharide (+7 hrs LPS); *x *axis) was plotted against the normalized ratio of +7 hrs LPS/unstimulated BMMs (*y *axis). Color of squares indicate the signal intensities are above (black) or below (gray) the background threshold.

The splicing array consisted of 1,717 oligonucleotide probes representing alternate transcripts arising from 106 TK. Probes were designed across splicing junctions as well as each exon. A representative set of intron probes for each TK was used as a negative control. Figure 1 shows the distribution of signals from junction probes, exon probes, and intron controls, demonstrating a high degree of signal specificity for the junction and exon probes. Primary bone-marrow derived macrophages (BMMs) were differentiated in the presence of colony stimulating factor 1 (Csf1) and profiled before and after exposure to the Gram-negative bacteria endotoxin lipopolysaccharide (LPS). We detected most of the predicted splice variants in either macrophage population (summarized in Table 1). The majority (1,445 out of 1,717 probes) from all 106 TKs were reliably expressed by macrophages in the unstimulated or LPS-activated state. Eighty-eight probes from 26 TKs were significantly induced after LPS exposure (*t* test, *P *< 0.05); as expected, these were primarily the inflammatory targets of Tlr signaling (and mediators of inflammation) such as chemokines and cytokines (TNF-α, ILs), as well as receptors, signal transduction molecules, and transcription factors. These data are consistent with the known inflammatory profiles of mouse macrophages after LPS exposure [10]. Thirty-nine probes from 13 TKs were significantly repressed (*t* test, *P *< 0.05) by LPS/Tlr4 signaling; these included transcript variants of receptors (Csf1r and Infar1), adapters (Toll interacting protein (tollip)), kinases (Irak4, Map3k1, Map3k7, Mapk8, Mapk14, phosphoinositide-3-kinase (Pik3)cg), and transcription regulators (activating transcription factor 2 (Atf2), DNA methyltransferase (Dnmt)1, small ubiquitin-related modifier (Sumo)) and effector molecules (serpin peptidase inhibitor, clade b (Serpinb3b)). These data concur with our previous arrays using representative probes for inflammatory gene targets [10].

A database with pathway map was built for ease of navigation through the Tlr signaling cascade, with TK numbers linking to a comparison of each transcript in the framework and a dynamic interphase with the FANTOM3 database. The genome viewer has been annotated with a gff track for each splicing probe on the array. Functional predictions from this data set are highlighted in the following sections, reviewing the impact on each part of the Tlr pathway, from receptor, signal transduction, and effector molecules.

### Alternate splicing impacts on receptor complexes

The genomic structure of the Tlr family in mice is characterized by a small number of exons (typically three), with the open reading frame (ORF) contained in the last exon. Alternate splicing of the 5'-untranslated region (UTR) is a common feature of this family in mouse and human isoforms, and arises from different transcriptional start sites (TSSs) as well as the internal splicing of UTR exons. Tlr3 transcripts with variable 5'-UTR were found to be differentially regulated on the splicing arrays (Additional data file 3). Although the functional significance of variable 5'-UTR length in this class of receptors is not understood, the use of different TSSs implies differential promoter usage and this, combined with modifications of UTR structure, presumably alters the stability and translational efficiency of the mRNA.

**Figure 2:**
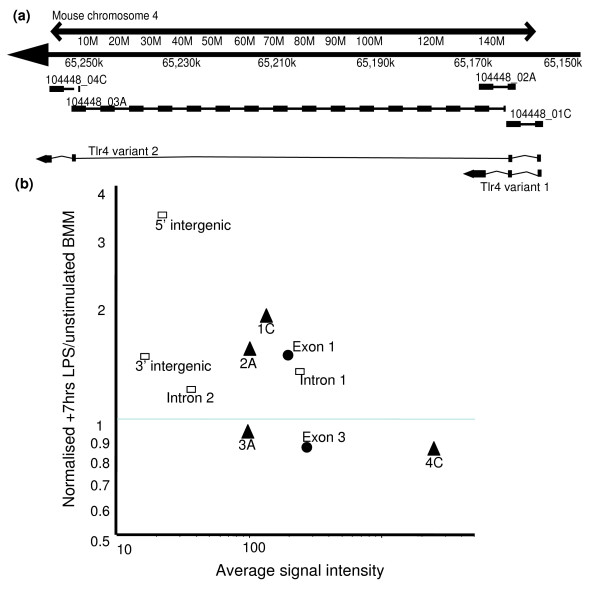
Murine Tlr4 encodes two variant transcripts: exon structure and expression of variant transcripts in BMMs. **(a) **Genome viewer tracks. Mouse Toll-like receptor Tlr4 maps to the minus strand of chromosome 4. Probe track indicates the exon junctions spanned by oligoprobes on the splicing array. Probe 1C spans the junction between exons 1 and 2. 2A spans the junction between exons 2 and 3, and is unique to the full-length Tlr4 variant. 3A and 4C span exons 2-4 and 4-5, respectively. Both probes detect the variant Tlr4 transcript. Transcripts are shown by exon-boxed cartoon. Variant 1 is a full-length Tlr4 transcript, variant 2 skips exon 3 and so lacks the transmembrane and cytoplasmic domains. **(b) **Scatter plot shows the distribution of expression detected by each probe. The average signal of each probe (unstimulated bone-marrow derived macrophages (BMMs) and BMMs subjected to seven hours of stimulation with lipopolysaccharide (+7 hrs LPS); *x *axis) was plotted against the normalized ratio of +7 hrs LPS/unstimulated BMMs (*y *axis). Junction probes are indicated by a triangle; intronic or intergenic controls are indicated by open boxes; and exon probes are indicated by black circles. The microarray detects expression of both Tlr4 transcripts in unstimulated and +7 hr LPS stimulated BMMs, and demonstrates a higher level of expression of the full-length variant.

Mouse Tlr4 (TK 104448) was the only member predicted to express a variable protein (Figure 2). The canonical receptor has three exons, with the ORF spanning exons 2 and 3, producing a 3.8 kilobase (kb) transcript and 836 amino acid transmembrane protein. The variant transcript (from RIKEN clone 4631423H05) skips exon 3 and splices onto exons 4 and 5 approximately 90 kb downstream of exon 2. The 1.8 kb transcript is predicted to encode a 154 amino acid secreted protein. This product does not appear to be conserved in humans at the transcript or genome level; human TLR4 has four reported variants, all of which alter the length of the extracellular leucine-rich repeat, although the functional significance of this has not been tested.

**Figure 3:**
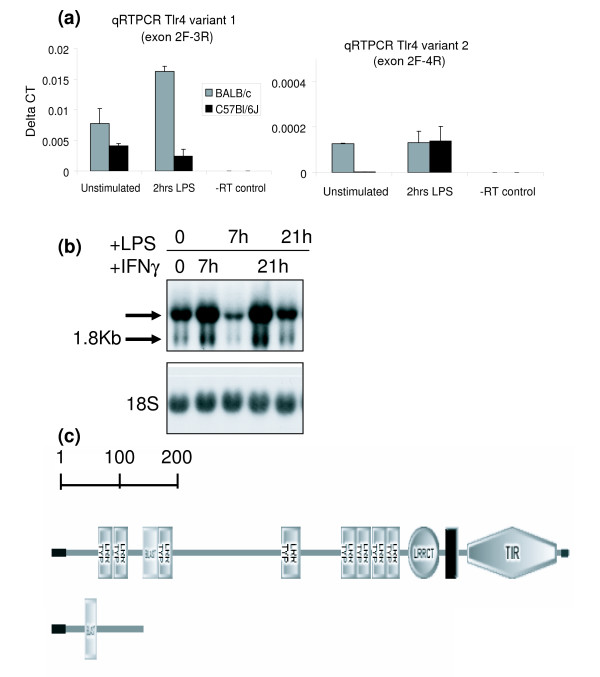
Validation of expression of Tlr4 transcripts by Northern blot and quantitative real-time-PCR. The expression of Toll-like receptor Tlr4 transcripts is detected by Northern blot and quantitative real-time (qRT) polymerase chain reaction (PCR), and is regulated in mouse bone-marrow derived macrophages by interferon (IFN) and lipopolysaccharide (LPS). Primary BMMs from two mouse strains (BALB/c and C57Bl/6J) were prepared and maintained overnight in the presence or absence of Csf1 (1 × 10^4 ^U/ml) before stimulation with IFN-γ (500 pg/ml) or LPS (10 ng/ml). **(a) **qRT-PCR expression of full Tlr4 (left) and truncated variant (right). Both transcripts were detected at higher levels in BALB/c BMMs. The expression of the truncated variant was 10-fold lower than the full-length variant in both mouse strains. Delta CT values were calculated from three replicates and normalized to a HPRT control primer set. **(b) **The expression of Tlr4 mRNA from C57Bl6/J BMM was detected by Northern blot using a ^32^P-labeled probe encoding part of the leucine rich repeat (LRR) domain, common to both variant Tlr4 transcripts. The membrane was stripped and re-probed with 18S ribosomal RNA as a loading control. The expression of both variants was induced by IFN and LPS. **(c) **SMART predictions of the protein domains found in the full-length (top) and truncated (bottom) Tlr4 transcripts. The variant transcript retains a signal peptide and leucine-rich domain, but it lacks the transmembrane and cytoplasmic domains.

Both variants of mouse Tlr4 are inducible by interferon-γ priming as well as LPS stimulation of primary mouse macrophages. Figure 3 demonstrates expression of the 3.8 and 1.8 kb Tlr4 variants by Northern blot analysis, splicing arrays, and quantitative real-time polymerase chain reaction (PCR) in C57Bl/6J and BALB/c mice. We confirmed the exon composition of the variants by designing primers that spanned exons 2-3 (3.8 kb variant) or exons 2-4 (1.8 kb variant) and testing the expression of Tlr4 in two different mouse strains - BALB/c and C57Bl6J - by quantitative real-time PCR.

The outcome of Tlr signaling is modified by the presence of interferon and Csf1 in the local environment. We therefore also examined the receptors for Csf1 (Csf1R; TK 167944) and the IFNAR subunits. Our computational pipeline successfully detected the alternatively spliced products of IFNAR1 (TK 177219) and IFNAR2 (TK 108218) TK subunits, and their expression was confirmed in the array dataset. These have been functionally characterized as dominant-negative modulators of the type I interferon response [12-14]. Similarly, a number of variants of the Csf1r have been identified from the FANTOM3 data set, including at least two that lack the catalytic cytoplasmic domain, one of which is predicted to be secreted by PSORT [21]. The expression of soluble receptors provides a 'ligand sink' that attenuates the dosage of cytokine available to local macrophages. Likewise, membrane tethered but catalytically dead variants may alter the capacity of a cell to respond to cytokines in the environment. This is important in an inflammatory context because Csf1 and type I interferons have been shown to prime and exacerbate the inflammatory response, and expression of dominant-negative receptors may antagonize this process.

### Alternate splicing of chaperones and adapters predicts variable scaffolds for signaling cascades

The most fundamentally conserved Tlr signaling pathway requires recruitment of Myd88 to the receptor complex [3]. Splicing of Myd88 has previously been reported, generating a short protein (Myd88s) that lacks the intermediate region between the Tir domain (which interacts with the Tlr) and the death domain (which interacts with downstream kinases). Myd88s was shown to repress inflammatory signals normally mediated through Myd88 [15].

Mice that lack Myd88 exhibit profound deficiencies in innate immune responses [22], but these same studies demonstrated that Myd88-independent signaling pathways were also important for effective innate immune function. Several adapter proteins are known to associate directly with Tlr in a Myd88-independent manner. These include Tollip; TIR domain-containing adapter protein (Tirap); TIR-containing adaptor molecule (Ticam)1 and Ticam2; JNK interacting protein (Jip)1, Jip2 and Jip3; and the rho family small GTP binding protein 1 (Rac1). We found variant transcripts for each of these adapter molecules, and confirmed their expression in naïve and LPS-activated macrophages on the splicing arrays.

**Figure 4:**
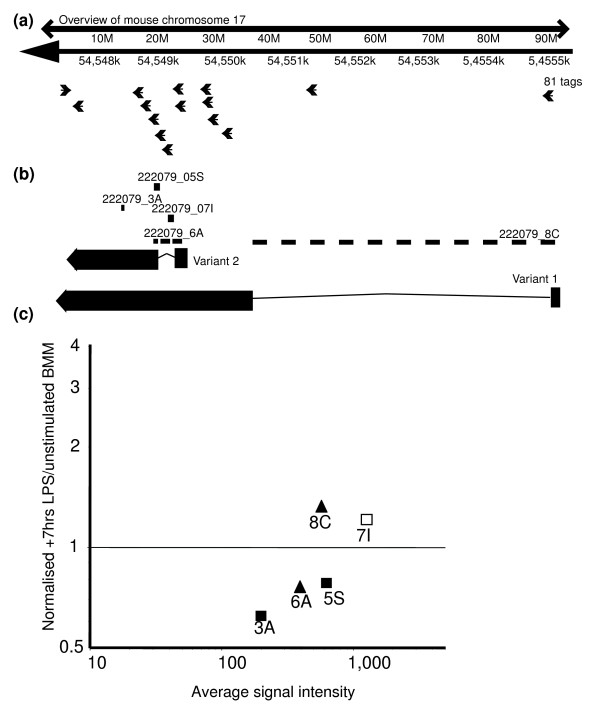
Variant transcript analysis of murine TIR-containing adaptor molecule (TICAM)1. **(a) **Genomic arrangement of murine TIR-containing adaptor molecule (Ticam)1 on the minus strand of mouse chromosome 17. TC (cap-analysis gene expression (CAGE) tag clusters) indicate transcriptional start sites (TSSs) from CAGE data (indicated by arrows). Two Ticam1 transcripts initiate from alternate TSSs: variant 1 from the major TSS (81 CAGE tags) and variant 2 from a cluster of CAGE tags between 54,549 K and 54,550 K. **(b) **The probe track indicates the exon junctions spanned by oligoprobes on the splicing array. Probe 8C spans exons 1-2, 7I lies in the region spliced between exons 1b and 2b, and 5S spans the intron/exon boundary of exon 2b. These probes are unique to variant 1. 6A spans the junction between exons 1b and 2b and is unique to variant 2. **(c) **Scatter plot shows the distribution of expression detected by each probe. The average signal of each probe (unstimulated bone-marrow derived macrophages (BMMs) and BMMs subjected to seven hours of stimulation with lipopolysaccharide (+7 hrs LPS); *x *axis) was plotted against the normalized ratio of +7 hrs LPS/unstimulated BMM (*y *axis). Junction probes are indicated by a triangle; intronic or intergenic controls are indicated by open boxes; and exon probes indicated by black squares. Both the full-length open reading frame (isoform 1) and the spliced 310 amino acid protein (isoform 2) are detected on the splicing array. CAGE data and splicing array expression analysis indicates that the full-length variant is more abundant.

We discovered transcript variants of Ticam1 (TK 222079) and Ticam2 (also known as Tram; TK 84178), which are intermediates in the interferon-response pathways initiated by Tlr3, Tlr4, and Tlr7. Ticam2 has an alternate TSS, resulting in a variable 5'-UTR length. Ticam1 and Ticam2 have ORFs that are encoded in the last exon of their respective frameworks, but internal splice sites in exon 2 of Ticam1 result in four alternate transcripts and two alternate proteins (Figure 4). The full-length (uninterrupted exon 2) transcript is 732 amino acids long. Transcripts initiating in this exon are spliced, and generate a 310 amino acid protein with a carboxyl-terminal truncation and containing only the TIR domain and proline-rich motifs. The truncated variant was expressed at lower levels (Figure 4c) than the full-length variant, and we predict that this novel protein variant may act as a dominant-negative in a similar manner to the truncated Myd88s.

Mapks and phosphoinositide-3-kinases (Pik3s) are recruited at several points in the Tlr signaling cascade. Jip3 is a Mapk-interacting protein that interacts directly with Tlr4 [23]; likewise Rac1 has been shown to recruit Pik3 through an interaction with Tlr2 [24]. Five variants of Jip1 and Jip3 have previously been reported [25] in mouse; we identified a sixth variant of each in the FANTOM3 dataset (Additional data file 1). Two variants of Rac1 have been reported in humans - isoform 1 is 192 amino acids and isoform 2 is 211 amino acids long [26,27]; we identified their mouse equivalents in the FANTOM3 data set. These scaffolding proteins function to recruit different partners of a kinase cascade. Alternate splicing of these adapter proteins has been shown by others to alter the interaction between scaffold and kinase, and so alter the interaction between kinases themselves and ultimately the signaling outcomes of that cascade [27,28].

### Alternate splicing modulates Iraks and their chaperones

The Irak family of receptor-associated kinases are important mediators of Tlr and IL-1 receptor signaling. Four members of the murine Irak family have been described, and functional studies have demonstrated regulation of Irak signaling by inhibitory isoforms. Two inhibitory splice variants of Irak1 have been functionally validated: Irak-s [29] and Irak1b [30]. Four splice variants of Irak2 have previously been reported, two of which inhibit Tlr4 signaling [16]. Irak3 (also known as Irak-m) is a dominant-negative member of the Irak family that also plays an important role in the repression of Tlr4 signaling [31]. Our data describe novel variants of Irak2 and Irak4 that are also predicted to be inhibitory, and are expressed in inflammatory macrophages, and further demonstrate variants of the chaperone proteins Tollip and Pellino.

The Iraks are important early signaling intermediates in Tlr and IL-1 cascades. We discovered novel isoforms of Irak2 (TK 181931) and Irak4 (TK 121381) in the FANTOM data set. Irak2e is a novel 151 amino acid protein generated by termination in a unique exon 4; the ORF retains only the death domain. Four protein variants of Irak4 are predicted. Irak4a is a 459 amino acid protein. Irak4b uses an alternate terminating exon, which resulted in a 453 amino acid protein with a variant amino-terminus. Irak4c, a 197 amino acid protein, terminated in exon 6 and retained only the death domains. Irak4d was a similarly truncated 195 amino acid protein resulting from cryptic splice site in exon 5. Irak4c and Irak4d are predicted to be dominant-negative variants.

**Figure 5:**
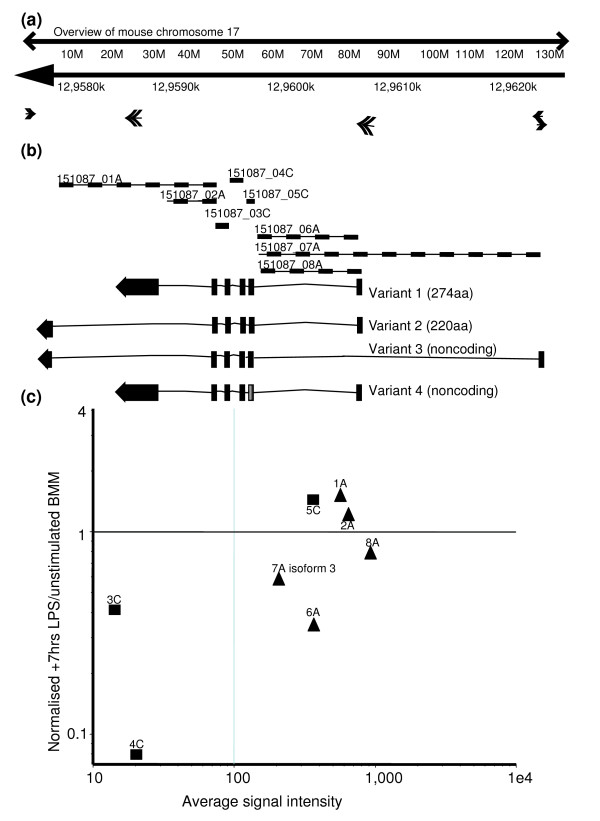
Variant transcript analysis of murine Toll interacting protein (Tollip). **(a) **Genomic arrangement of murine Toll interacting protein (Tollip) on the minus strand of mouse chromosome 7. TC (cap-analysis gene expression (CAGE) tag clusters) indicate two transcriptional start sites (TSSs): variants 1, 2 and 4 are from the major TSS at 129,607 K and variant 3 is from a cluster of CAGE tags (indicated by arrows) at 129,623 K. **(b) **The probe track indicates the exon junctions spanned by oligoprobes on the splicing array. Variant transcripts are indicated by boxed exon cartoon. Variant 1 is a 274 amino acid protein initiating from exon 1B. Variant 2 is a 220 amino acid dominant-negative protein that skips exon 6 and so lacks the carboxyl-terminal CUE domain. Variant 3 initiates from exon 1A and lacks an open reading frame (ORF). Variant 4 contains a cryptic splice acceptor in intron 3 that disrupts the ORF, resulting in a noncoding transcript. **(c) **Scatter plot shows the distribution of expression detected by each probe. The average signal of each probe (unstimulated bone-marrow derived macrophages (BMMs) and BMMs subjected to seven hours of stimulation with lipopolysaccharide (+7 hrs LPS); *x *axis) was plotted against the normalized ratio of +7 hrs LPS/unstimulated BMM (*y *axis). Junction probes are indicated by a triangle and exon probes are indicated by black squares. Probe 1A measured the expression of the alternate terminating exon associated with variant 2. Probes 7A and 8A measured the expression of variants 3 and 4, respectively. Probe 6A (variant 1) was significantly repressed after LPS treatment (*P *< 0.05).

The FANTOM3 data set contains a novel 220 amino acid Tollip (TK 151087) isoform generated from an alternate terminating exon that lacks the amino-terminal CUE domain, as illustrated in Figure 5. Tollip is a 274 amino acid protein that interacts with the ubiquitination machinery through an amino-terminal CUE domain, and has also been shown to interact directly with Tlr2 and Tlr4. Tollip inhibits Irak signaling by targeting it for ubiquitination [32]. This domain is important for the interaction of Tollip with Tom1 and the ubiquitination pathway [33], and so an amino-terminal truncated variant was predicted to exacerbate inflammatory signaling through Tlr and Irak pathways. Both protein encoding isoforms were expressed by macrophages on the splicing array. The full-length 274 amino acid isoform was slightly but significantly repressed at seven hours of LPS treatment (fold change, *t* test, *P *< 0.05).

Human Tollip is also highly alternatively spliced, generating several coding and noncoding variants. The full-length human Tollip is a 274 amino acid protein. Alternate usage of exons 2 and 3 generate a noncoding RNA and a 213 amino acid variant, respectively. Similarly, we detected two transcripts with no obvious ORF expressed from the mouse Tollip framework. One was from upstream exon 1A, which is expressed approximately ten-fold less than the transcripts originating from exon 1B. The second noncoding transcript originated from exon 1B and spliced onto a cryptic splice acceptor in intron 3, interrupting the ORF.

Pellino2 (Peli2, TK 104834) is a scaffolding protein that interacts with Irak1 and Irak4. Three Peli2 isoforms are generated from four transcript variants. Peli2a is an 438 amino acid protein; Peli2b is a 319 amino acid protein and is encoded by two different transcripts: the first initiates at exon 1 but skips exons 2 and 3, and the second initiates at exon 3b (unique to this variant). Peli2c is an amino-terminal truncated 155 amino acid protein that initiates at exon 1 and terminates at exon 3a (unique to this transcript), and is lacking the Pellino domain. All three variants are detected in macrophages on the splicing array, and by cap-analysis gene expression (CAGE) tag analysis. Strelow and colleagues [34] first characterized Peli2, and identified several protein variants by western analysis, including one lacking the amino terminus, but these were assumed to be post-translational modifications of Peli mediated by Irak. Our data indicate that this truncated peptide is most likely Peli2c, generated by alternate splicing of the Peli2 TK, and generating a dominant-negative protein that lacks the Pellino domain.

These data demonstrate further regulation of Irak signaling, indicating that this is tightly controlled at a transcriptional level by the production of numerous dominant negative isoforms, as well as chaperones that are predicted to alter the location and association of Irak and its targets.

### Alternate splicing of the Mapk and Pik3 pathways alter inflammatory signaling in macrophages

The Kyoto Encyclopaedia of Genes and Genomes identified 30 kinases that intersected the Tlr pathway, from which we predicted that alternate transcription generated total of 72 novel kinases available to Tlr signaling in macrophages. The Mapk and Pik3 pathways in particular are integral to Myd88 dependant and independent signaling outcomes. The Mapk cascade diversifies inflammatory signaling, whereas Pik3 is thought to temper the severity of the acute signal from Tlr (for review, see [35]).

**Figure 6:**
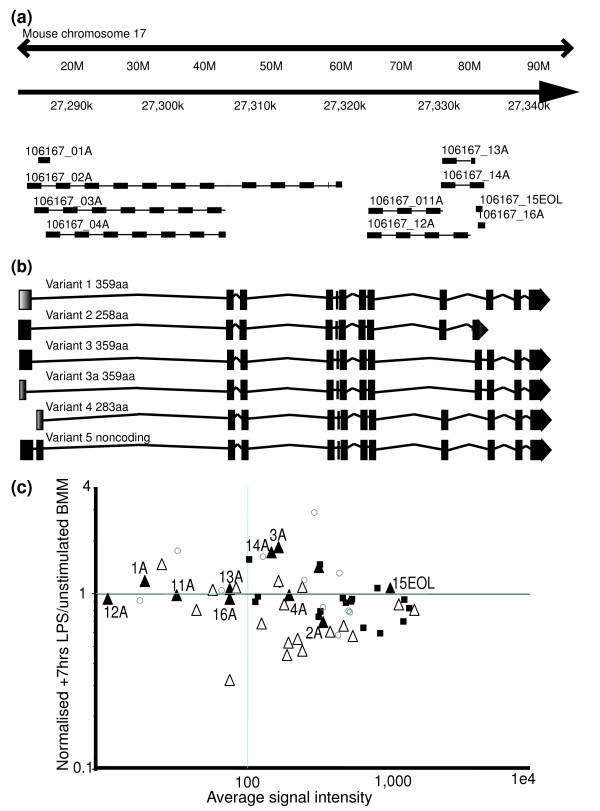
Murine Mapk14/p38-α encodes four variant proteins: exon structure and expression in BMMs. **(a) **Genomic arrangement of murine mitogen-activated protein kinase (Mapk)14 on the plus strand of mouse chromosome 17. The probe track indicates the exon junctions spanned by oligoprobes on the splicing array. **(b) **Variant transcripts are indicated by boxed exon cartoon. Variants 1 and 3 are 359 amino acid proteins that alternate exon 10 (variant 1) or exon 11 (variant 3). Piccolo (variant 2) is a 258 amino acid dominant-negative isoform. Variant 4 originates from a novel transcription start site (TSS) with a predicted 283 amino acid open reading frame (ORF) lacking the ATP-domain. Variant 5 included a novel exon that interrupts the ORF. **(c) **Scatter plot shows the distribution of expression detected by each probe. The average signal of each probe (unstimulated bone-marrow derived macrophages (BMMs) and BMMs subjected to seven hours of stimulation with lipopolysaccharide (+7 hrs LPS); *x *axis) was plotted against the normalized ratio of +7 hrs LPS/unstimulated BMM (*y *axis). Open triangles indicate constitutive junction probes, black triangles indicate alternate junction probes, and exon probes are indicated by black squares. Probe 3A measures the inducible expression of variant 4 after LPS treatment (*P *< 0.05). Probes 13A and 15EOL measure the expression of variant 2 (piccolo), which was low compared with variant 1 or 4 (measured by probe 14A). Probe 12A confirms a low expression of variant 3 in macrophages.

Mapk14/p38 (TK 106167) is emerging as an important modulator of macrophage signaling in response to a diverse set of PAMPs. Three protein variants of p38 have been described to date: isoforms 1 and 3 are 359 amino acid proteins with the variable inclusion of exon 10 (isoform 1) or exon 11 (isoform 3). Human EXIP (mouse piccolo), the carboxyl-terminally truncated Mapk14 258 amino acid variant, has been shown to interact directly with TOLLIP and Irak to downregulate signaling [36]. Our data predicted a fourth, amino-terminally truncated Mapk14 283 amino acid variant initiated from a novel exon 1B, and confirmed the expression of isoforms 1, 3, and 4 in macrophages. Figure 6 demonstrates the complex transcription arising from the Mapk14 TK, and profiling indicated that expression of Mapk14 variants is differentially regulated, with isoforms 1 and 3 repressed by LPS exposure of mouse macrophages.

**Figure 7:**
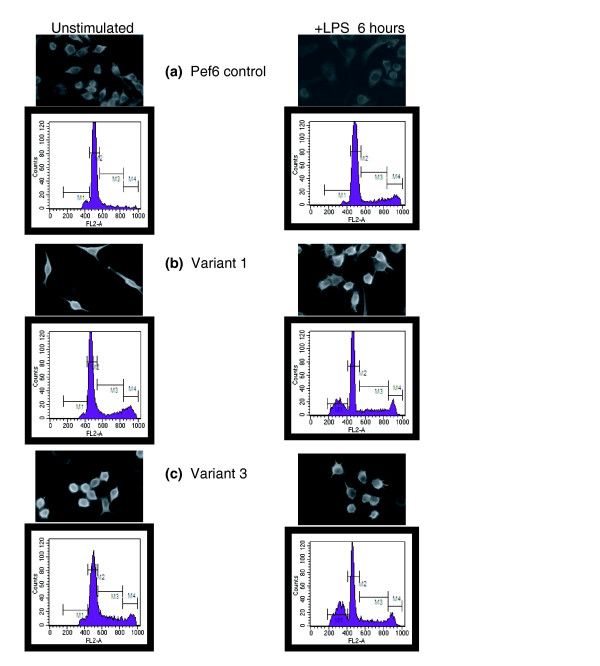
Mapk14/p38-α isoforms differentially alter macrophage survival. Raw264.7 cells were transfected with **(a) **pef6 vector only, **(b) **mitogen-activated protein kinase (Mapk)14 variant 1, and **(c) **Mapk14 variant 3. Each panel illustrates the transfectants before (left panels) and after (right panels) 6 hours of exposure to lipopolysaccharide (LPS; 10 ng/ml). Fluorescence activated cell sorting (FACS) analysis showed the percentage of cells in each stage of the cell cycle: apoptosis (A), G_1_, S, and G_2 _phases. The FACS findings in panel a were as follows: unstimulated pef6 control - A 2.4, G_1 _69, S 17.6, and G_2 _10.9; and +LPS - A 3.1, G_1 _78.6, S 9.2, and G_2 _9.4. In (b) the FACS findings show that Mapk14 isoform 1 lowered inflammatory cell survival: FACS before - A 3.5, G_1 _74, S 12, and G_2 _10.6; and +LPS - A 20.5, G_1 _59.5, S 12, and G_2 _8.2. In (c) the FACS findings show that Mapk14 isoform 3 over-expression alters cell survival in unstimulated and inflammatory cells: FACS before - A 9, G_1 _65, S 8, and G2 18; and +LPS - A 32.5, G_1 _47.5, S 7, and G_2 _13.

Mapk14 is central to inflammatory signaling, and the functional validation of variants of the Mapk14 framework illustrates the potential for regulation of signal transduction by other Mapk and Pik3 variants. We overexpressed isoforms 1 and 3 of Mapk14 in RAW264.7 cells to evaluate the impact of these proteins on inflammatory activation and survival of macrophages (Figure 7). Overexpression of isoform 3 led to an overtly activated macrophage phenotype even in unstimulated cells, whereas over-expression of isoform 1 did not change the morphology of the RAW cells. The survival of RAW264.7 cells exposed to LPS was modified by over-expression of both variants (Figure 7b); however, we noted that over-expression of isoform 3 led to cell cycle arrest in RAW264.7 prior to LPS exposure.

Signal transduction in the Tlr cascade has great potential to be similarly regulated at a genomic level through variation in other kinases. Of the 72 variant proteins generated from the 30 Mapk and Pik3 TKs examined here, 15 were predicted to generate dominant-negative isoforms (Additional data file 1), and it is a reasonable supposition that a significant proportion of these will act competitively to alter inflammatory signaling and macrophage differentiation/survival.

### Alternate splicing generates a large cohort of transcription factors and chromatin modifiers

Signal transduction by Tlr receptors culminates in the activity of nuclear factor-κB (via Irak/Traf6), Ap-1/Erk/Elk (via Mapk), Irf (via Ticam) and Stat (via Ifnar) [37]. Thirteen transcriptional regulators, including Ikbkb, Ikbke and Ikbkg, were predicted to generate 49 variant proteins. The Atf2 framework alone generated 18 variants.

**Figure 8:**
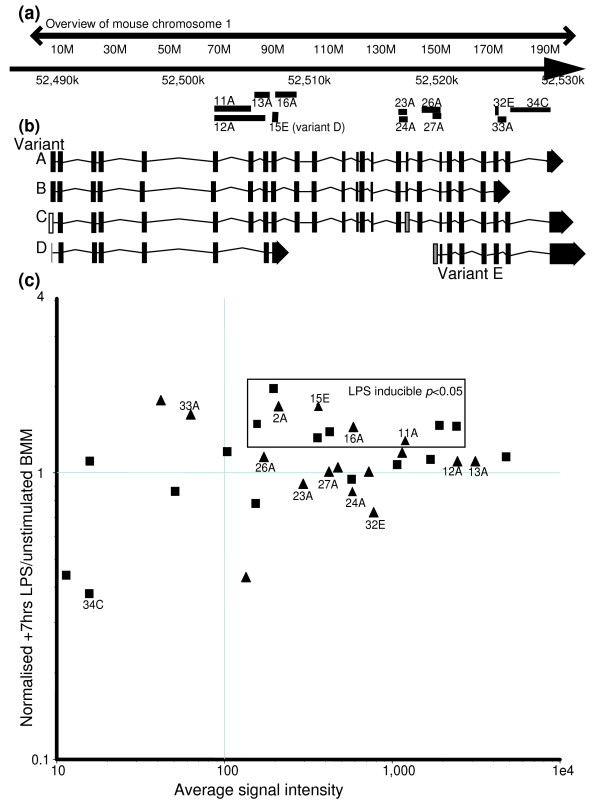
Murine Stat1 encodes four variant proteins: exon structure and expression of variant transcripts in BMMs. **(a) **Genomic arrangement of murine Stat1 on the plus strand of mouse chromosome 1. Probe track indicates the exon junctions spanned by oligoprobes on the splicing array. **(b) **Variant transcripts are indicated by boxed exon cartoon. Stat1a is a 749 amino acid protein initiating from exon 1A. Stat1b is 712 amino acids, terminates at exon 23, truncating carboxyl-terminal 37 amino acids. Isoform c is 755 amino acids with alternate initiation at exon 1B (open box), and the insertion of an additional 6 amino acids (VFVPFQ) in the DNA binding domain by using cryptic splice site upstream of exon 17 (grey exon box). Isoform d is a 177 amino acid protein that skips exon 7 and terminates at exon 9. There are at least four noncoding transcripts observed, initiating from exons 1, 19, and 22 (not shown). **(c) **Scatter plot shows the distribution of expression detected by each probe. The average signal of each probe (unstimulated bone-marrow derived macrophages (BMMs) and BMMs subjected to seven hours of stimulation with lipopolysaccharide (+7 hrs LPS); *x *axis) was plotted against the normalized ratio of +7 hrs LPS/unstimulated BMM (*y *axis). Triangles indicate junction probes and exon probes are indicated by black squares. Differential expression was determined using a *t* test cutoff of *P *< 0.05.

We identified a number of novel Stat isoforms in the FANTOM data set, including four Stat1 isoforms (TK100600), two Stat2 (TK 98057) isoforms, and three Stat3 isoforms (TK 108218). Each of these frameworks had variant transcripts induced by LPS-stimulation of macrophages, as measured on the splicing arrays. The data for Stat1 are shown in Figure 8. Stat1 is known to produce alpha (749 amino acid) and beta (712 amino acid) isoforms in human and mouse [38]. Two novel isoforms are predicted in the FANTOM3 dataset - a 755 amino acid and a 177 amino acid isoform. CAGE tag analysis (Figure 8a) of the Stat1 promoter indicated that the novel 755 amino acid isoform initiation at exon 1B was most highly expressed in adipocytes, and infrequently used by macrophages. This variant inserts an additional 6 amino acids (VFVPFQ) in the DNA binding domain by using cryptic splice site upstream of exon 17, and indeed this probe was detectable but not highly expressed in the macrophage populations screened on the splicing arrays (Figure 8).

Three of the four Stat1 isoforms were expressed in macrophages, and one of these - Stat1a - was differentially regulated by seven hours of exposure to LPS. Figure 8 shows data from the splicing arrays that indicated differential expression of Stat1 isoforms after LPS challenge of macrophages. Junction probes spanning alternate (A) and constitutive (C) exons 6-7 (probe11A), 6-8 (probe 12A), and 7-8 (probe 13A; Figure 7) showed that the 177 amino acid isoform lacking exon 7 was highly expressed in macrophages, but was not regulated at seven hours by Tlr4/LPS signaling. This isoform lacks the DNA binding and SH domains, retaining just the first STAT domain, and is predicted to be a dominant-negative protein.

The array data indicate that mRNA of the alpha but not the beta isoform is LPS/Tlr4 induced. The isoforms containing exon 7 and most of the constitutive exons were induced by LPS (*t* test, *P *< 0.05). The terminating exon probe for the beta isoform was highly but not differentially expressed at seven hours of LPS treatment, whereas the probes unique to the alpha isoform were expressed at a lower level, but the expression of these was higher in the LPS stimulated state.

Macrophages respond rapidly at a transcriptional level to PAMPs, and this occurs at the level of chromatin remodeling as well as transcription factor binding. Several chromatin modifiers, histone deacetylases (Hdac), MYST histone acetyltransferases (Myst), DNA methyltransferases (Dnmt), and the small ubiquitin-related modifiers (Sumo) were found to generate a large number of alternatively transcribed products. Interestingly, although macrophages expressed most of these variants, isoforms of Dnmt1 and Sumo were downregulated by LPS signaling.

## Conclusion

The impact of alternate transcription on innate immune signaling has been hinted at by several studies on individual dominant-negative variants of key signaling molecules, including Ifnar2, Myd88, and Irak1 and 2 [14-16,29]. We have surveyed the transcripts arising from TKs for 107 members of the Tlr signaling pathway in mouse, and propose that the production of protein variants in general, and of dominant-negative proteins in particular, is a widespread mechanism for the regulation of inflammation.

The complexity of alternate splicing per TK increased as we progressed from receptor complex to transcriptional regulator. The Tlr receptors were typically encoded from a single exon ORF, arguing that diversification of this receptor family has occurred through gene expansion rather than alternate splicing of protein products. The exception was Tlr4, for which we described a novel transcript predicted to encode a secreted Tlr4 isoform. Downregulation of the Tlr4 receptor complex by soluble CD14 (produced from a post-translational cleavage) has been demonstrated, as well as interactions with Tlr1 and Rp105 [39]. Further analysis of the 1.8 kb variant is necessary to determine whether alternate splicing of Tlr4 itself adds another inhibitory mechanism to the Tlr4 receptor complex. We found that at least nine of the adapter molecules and 16 of the kinases in this pathway alone produced multiple, variant protein products through alternate splicing. Of these, we demonstrated the proapoptotic effects of Mapk14 variants on LPS-stimulated macrophages. Alternate transcription of chromatin remodeling complexes and transcription factors could generate a large number of variant proteins, and the expression of these variants was regulated by LPS/Tlr signaling. The inflammatory effector molecules released by Tlr signaling - interleukins, chemokines and cytokines - act to amplify rapidly the inflammatory signal, by recruiting and priming immune cells. As the repertoire of signaling molecules increased at each step in the Tlr cascade, we propose a concomitant increase in the repertoire of signaling events. Importantly, the expression of dominant-negative proteins for many key signaling molecules provides the immune system with opportunities to resolve inflammation.

A healthy innate immune system has the capacity to generate a broad repertoire of responses to combat infectious disease. Alternate transcription of Tlr pathways is just one mechanism of generating a combinatorial expansion of possible signaling outcomes, including repression of signaling at multiple points in the cascade. Why are so many points of control necessary? The flip side to these inflammatory pathways is susceptibility to inflammatory diseases. Uncontrolled inflammation leads to tissue damage and sepsis in an acute infection, and the Tlr signaling cascades have been linked to chronic inflammatory diseases such as atherosclerosis, arthritis, and pulmonary diseases. The role of variant signaling proteins in the pathogenesis of these diseases is yet to be explored; this study indicates that they are likely to provide new ways to target the inflammatory process.

## Materials and methods

### Transcript framework assembly and splicing predictions

The computation pipeline has been described in detail elsewhere [11]. Briefly, alternate transcript and peptide predictions were collected from the FANTOM3 and public data sets. Transcript frameworks of starts, ends, and exon boundaries were compiled for each cluster of transcripts. Domain predictions from InterPro for each alternate transcript were mapped to genomic location and are available as gff tracks (Additional data file 2). Predictions of membrane topology and presence of a signal peptide were generated using PHOBIUS [40], and those predicted as containing a signal peptide were analysed using PSORTII [21]. Gff tracks for splicing array probes and CAGE tissue expression have been made available to support the expression analysis.

### Splicing array expression profiling

Custom oligonucleotide microarrays were obtained from Combimatrix Corp (Mukilteo, WA, USA). The probes were designed by the manufacturer's automated pipeline to a length of 36 bases with 18 bases either side of the splice site. Probes were also designed manually for control genes with lengths ranging from 35 and 40 bases. All probes were designed as antisense probes, for detection of directly labeled mRNA.

*In situ *synthesized oligonucleotide arrays (Combimatrix Corp.) were designed to screen the expression of alternate transcription from these frameworks. The splicing array consisted of 1,717 oligonucleotide probes representing alternate transcripts arising from 106 TK. Probes were designed across splicing junctions, as well as each exon. A representative set of intron probes for each TK were used as negative controls. Figure 1 shows the distribution of signals from junction probes, exon probes, and intron controls, demonstrating a high degree of signal specificity for the junction and exon probes. Primary BMMs were differentiated in the presence of Csf1 and profiled before and after exposure to the Gram-negative bacteria endotoxin LPS.

Untreated and LPS treated (seven hours) primary BMMs were prepared as previously described [10]. RNA was extracted using QIAGEN RNeasy (for total RNA) followed QIAGEN oligotex resin (QIAGEN, Hilden, Germany). Two milligrams of polyA+ RNA was labelled directly with Cy3 or Cy5 using the Kreatech ULS labelling system (Kreatech Biotechnology, Amsterdam, Netherlands). Four pair-wise comparisons were performed, with a dye swap included for half of the experiments. Overnight hybridizations and data extraction was performed in accordance with the manufacturer's instructions. All data was Lowess normalized and visualized using Genespring7.2 (Silicon Genetics Inc., Agilent Technologies, Palo Alto, CA, USA). All expression data are available via GEO (accession number: GSE4044) or can be visualized via IMB Signet (Silicon Genetics Inc.) [41].

### Northern analysis

RNA samples were electrophoresed using denaturing formaldehyde agarose gels. Samples contained 20 μg RNA in 1 × MOPS (3-(N-morpholino)-propanesulfonic acid), 5.9% (weight/vol) formaldehyde, and 50% deionized formamide. An RNA marker (0.24-9.5 kb RNA ladder; Invitrogen, Carlsbad, CA, USA) was prepared, electrophoresed, and detected according to manufacturer's instructions for approximate size determination of RNA bands. The probe used for mouse *tlr4 *mRNA detection was amplified by PCR, and encoded part of the leucine-rich repeat domain (forward: 5'-AGAGAATCTGGTGGCTGTGG-3'; reverse: 5'-TCAACCGATGGACGTGTAAA-3'). The labeled probe was boiled for 5 minutes at 100°C before hybridization. 18S ribosomal RNA was detected using the oligonucleotide probe 5'-CATGGTAGGCACGGCGACTACCAT-3', as reported previously [42].

### Real-time PCR

cDNA levels of all genes and the internal control gene, murine hypoxanthine-guanine phosphoribosyl transferase (*hprt*), were estimated by quantitative PCR with the SYBR Green kit (Applied Biosystems, Foster City, CA, USA), gene-specific primers and an ABI Prism 7000 Sequence Detection System (Applied Biosystems). Threshold cycle values were calculated from amplification plots and gene expression was expressed relative to the control gene *hprt*. Gene-specific primer pairs were designed using Primer3 software [43,44]. Primers used were as follows: Tlr4 exon 2F tctgagcttcaaccccttg, Tlr4 exon 3R tgccatgccttgtcttca, and Tlr4 exon 4R gcctcggaggatttggat.

## Additional data files

The following additional data are included with the online version of this article: A csv file showing each transcript variant for the Tlr pathway, the splicing array probe sequences, and their genomic coordinates (Additional data file 1); a gff track of each splicing array probe for annotation of genome viewers (Additional data file 2); a text file showing all of the raw data from the four replica splicing array experiments (Additional data file 3); and a text file showing the Tlr database data, which may also be visualized and searched from our macrophages website [44] (these data include the full analysis of each predicted variant protein and links to a genome viewer with probe tracks overlayed as gff; Additional data file 4).

## Supplementary Material

Additional File 1 A csv file showing each transcript variant for the Tlr pathway, the splicing array probe sequences, and their genomic coordinates. Click here for file

Additional File 2 A gff track of each splicing array probe for annotation of genome viewers. Click here for file

Additional File 3 A text file showing all of the raw data from the four replica splicing array experiments. Click here for file

Additional File 4 A text file showing the Tlr database data, which may also be visualized and searched from our macrophages website [44] (these data include the full analysis of each predicted variant protein and links to a genome viewer with probe tracks overlayed as gff). Click here for file

## Figures and Tables

**Table 1:** Summary of splicing microarray data: unstimulated macrophages versus macrophages subjected to seven hours of LPS stimulation

	Expressed unstimulated macrophages	Expressed macrophages + 7 hr LPS	Variants repressed by LPS/Tlr4 (*t* test *P *< 0.05)	Variants induced by LPS/Tlr4 (*t* test, *P *< 0.05)
Transcript framework	107	106	13	26
Total number of probes	1,404	1,376	39	88
Receptors	16 TK		Csf1r, Ifnar1	Tlr3, Cd86, Clesf9
Adapters	13 TK		Tollip	Rac2
Signal transducers	30 TK		Irak4, Map3k1, Map3k7, Mapk14, Pik3cg, Serpinb3b	Pde4a, Pik3cd, Tbk1
Transcription factors	29 TK		Atf2, Dnmt1, Sumo	Nfkbia, Nfkb1, Nfkb2, Ikbke, Irf1, Stat1, Stat2, Stat3, Sin3a
Inflammatory effectors	17 TK		Serpinb3b	Ccl3, Ccl4, Ccl5, Cxcl10, Cxcl9, Il12b, Il1b, Il6, Casp8, Tnf-α

The splicing array consisted of 1,717 oligonucleotide probes representing alternate transcripts arising from 108 TK. The experiment was repeated four times, with dye-swap. LPS, lipopolysaccharide; TK, transcriptional framework; Tlr, Toll-like receptor.
